# Supplementary material for: DMRforPairs: identifying Differentially Methylated Regions between unique samples using array based methylation profiles
Source: BMC Bioinformatics. 2014 May 15;15:141. doi: 10.1186/1471-2105-15-141 (PMC4046028; doi:10.1186/1471-2105-15-141)
Supplement: Additional file 2 — DMRforPairs output for the comparison of A431-MCF7 and NA17018-NA17105. Please start from the HTML files in each folder. Available via the BMC Bioinformatics website. [file 1471-2105-15-141-S2.zip › 1394847754114233_MOESM2_ESM/A431_MCF7/figures/19966.pdf]

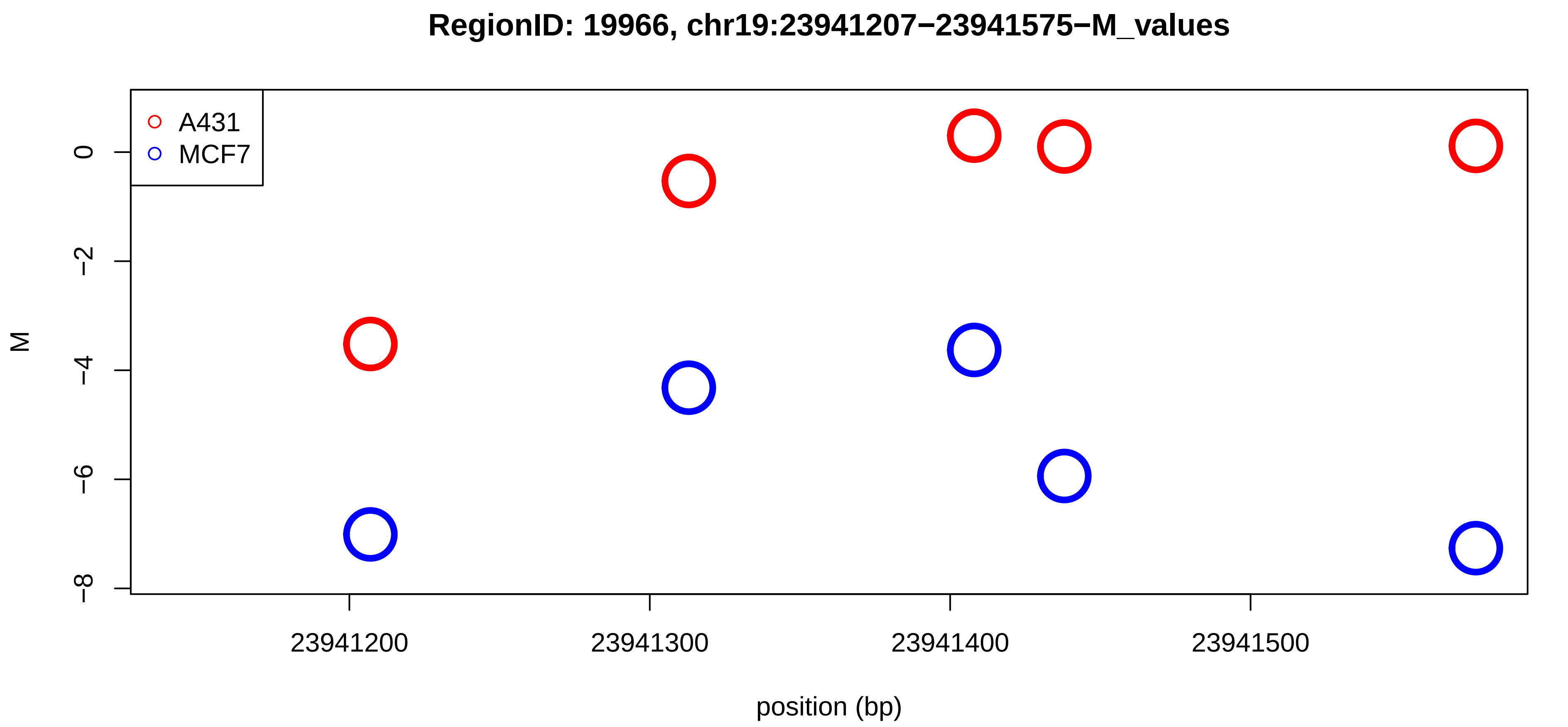
RegionID: 19966, chr19:23941207−23941575−M_values
A431
MCF7
M
0
−2
−4
−6
−8
23941200
23941300
23941400
23941500
position (bp)

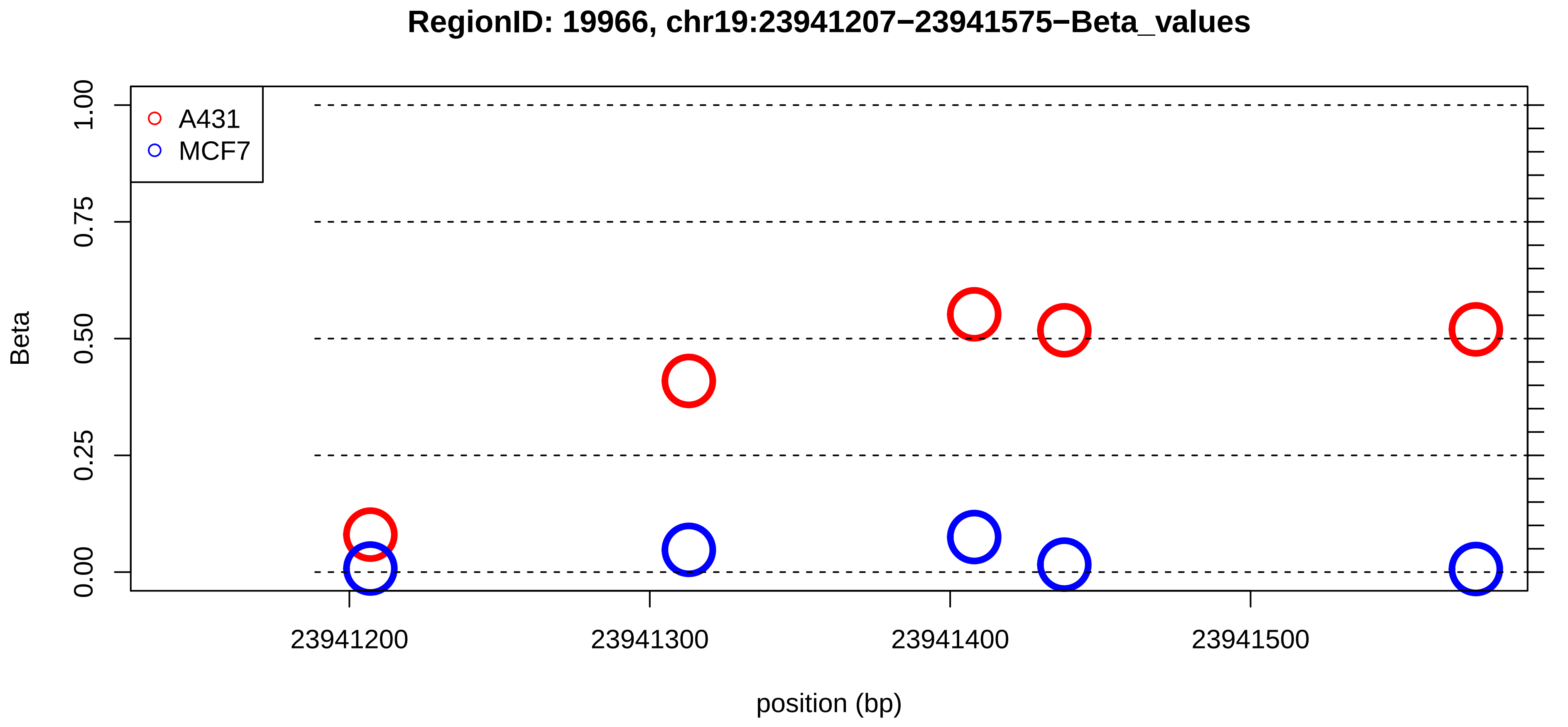
RegionID: 19966, chr19:23941207−23941575−Beta_values
A431
MCF7
Beta
1.00
0.75
0.50
0.25
0.00
23941200
23941300
23941400
23941500
position (bp)

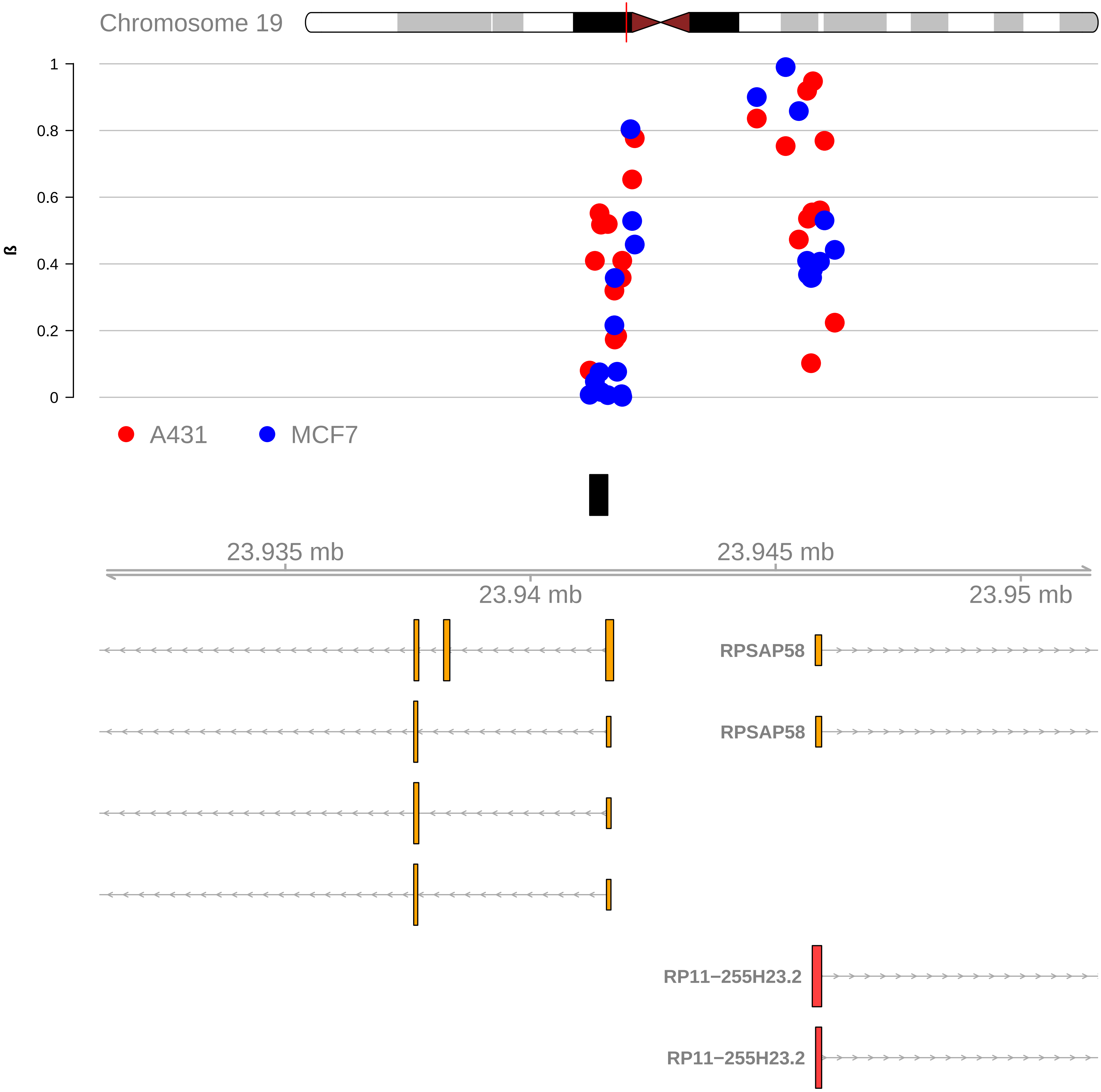
Chromosome 19
1
0.8
0.6
0.4
0.2
0
β
A431
MCF7
23.935 mb
23.945 mb
23.94 mb
23.95 mb
RPSAP58
RPSAP58
RP11−255H23.2
RP11−255H23.2
